# Perceived Social Support and Life Satisfaction in Infertile Women Undergoing Treatment: A Moderated Mediation Model

**DOI:** 10.3389/fpsyg.2021.651612

**Published:** 2021-05-28

**Authors:** Xiying Chu, Yaoguo Geng, Ruiping Zhang, Wenjing Guo

**Affiliations:** ^1^The Second Affiliated Hospital of Zhengzhou University, Zhengzhou, China; ^2^School of Education, Zhengzhou University, Zhengzhou, China; ^3^School of Marxism, Zhengzhou University, Zhengzhou, China

**Keywords:** perceived social support, life satisfaction, self-compassion, infertility self-efficacy, infertile women

## Abstract

Perceived social support is positively related to life satisfaction in infertile women. Whereas, the underlying mechanism of this relationship is unclear. The present study aimed to investigate whether self-compassion mediated the relationship of perceived social support with life satisfaction and whether infertility self-efficacy moderated the relationship between perceived social support and self-compassion in infertile women. A total of 290 infertile women in mainland China undergoing treatment completed an online survey assessing perceived social support, life satisfaction, self-compassion, and infertility self-efficacy. The results supported the mediation model that perceived social support was associated with life satisfaction via self-compassion. Besides, infertility self-efficacy moderated the relationship between perceived social support and self-compassion. Specifically, perceived social support displayed a stronger predictive effect on self-compassion when infertile women had higher level of infertility self-efficacy.

## Introduction

### Perceived Social Support, Self-Compassion, and Life Satisfaction in Infertile Women

Infertility is defined as the failure to achieve a successful pregnancy after 12 or more months of regular and unprotected sexual intercourse (Practice Committee of the American Society for Reproductive Medicine., 2008). There are two types of infertility: primary infertility and secondary infertility. Primary infertility means never having been pregnant. And secondary infertility is defined as having been pregnant at least once before meeting the criteria for infertility. In addition, Infertility may stem from an issue from male, female, or combined factors from male and female (Izzo et al., 2015). Infertility can lead to a decrease of the quality of life in both men and women. Whereas, women are more affected than men (Link and Darling, 1986; Schanz et al., 2011; Masoumi et al., 2016). But not all infertile women experience low life satisfaction. A positive association between perceived social support and life satisfaction has been found in infertile women in Poland and Italy, suggesting that perceived social support may improve life satisfaction in infertile women (Dembińska, 2016; Kiesswetter et al., 2020). Perceived social support broadly refers to the perception that one has a reliable social network to turn to and is cared for in times of need (Taylor, 2011). Individuals can perceive social support from multiple sources, such as family, friends, and significant others (Taylor, 2011). So far, the relationship between perceived social support and life satisfaction has not been examined in infertile women in mainland China. Moreover, it remains unclear how and why perceived social support was associated with life satisfaction in infertile women.

Self-compassion is an emotional positive self-attitude in the face of suffering or personal failure. It entails three basic components: (a) self-kindness–being kind and understanding to oneself in instances of pain or failure rather than being harshly self-critical, (b) common humanity–perceiving one’s painful experiences or failure as part of the larger human experience rather than seeing them as separating and isolating, and (c) mindfulness–holding painful thoughts and feelings in balanced awareness rather than over-identifying with them (Neff, 2003a). Emotional-approach coping strategy refers to the process by which individuals make effortful attempts to maintain awareness of, explore and understand their emotions (Stanton et al., 1994, 2000). According to Neff (2003a), self-compassion can be viewed as a useful emotional-approach coping strategy. In the process of self-compassion, painful or distressing feelings are approached with kindness, understanding, and a sense of shared humanity. Therefore, negative emotions are transformed to a more positive feeling state, allowing a clearer understanding of one’s immediate situation and the adoption of actions to change oneself and/or the environment in appropriate and effective ways (Neff, 2003a). Although limited attention has been given to self-compassion in studies of infertile individuals, self-compassion has been found to be a coping strategy used by infertile women and was positively associated with subjective well-being. Higher subjective well-being was defined as higher life satisfaction, more positive effect and less negative effect (Emmons and Diener, 1985). In a qualitative study conducted in Hong Kong, self-compassion and religion were found to be coping strategies used by infertile women (Tiu et al., 2018). Infertile women could be self-compassionate in the face of the psychological distress caused by infertility. In particular, they attempted to overcome the negative emotions and admit openly the pain. Furthermore, they went through the suppressed pain and tried to take up a more positive attitude toward infertility (Tiu et al., 2018). In a cross-sectional study, Raque-Bogdan and Hoffman (2015) found in infertile women mainly from the United States, Canada, and the United Kingdom that self-compassion showed a positive correlation with subjective well-being. These findings suggested infertile women may use self-compassion to manage their negative thoughts and feelings related to infertility, leading to improved subjective well-being. The culture of Hong Kong is similar to that of mainland China. Therefore, self-compassion may be a coping strategy for infertile women in mainland China, too. To our knowledge, the relationship between self-compassion and life satisfaction among infertile women in mainland China has not been examined.

As a useful emotional-approach coping strategy, self-compassion may be a mediator between perceived social support and subjective well-being in infertile women. Some researchers proposed that an important aspect of social support is it’s impacts on the coping strategies individuals adopt under stress. For instance, Lazarus and Folkman (1984) defined resources including social support as what an individual draws on to cope and argued such resources precede and affect coping. Analogously, social support was considered as a source of coping assistance by Thoits (1986). In line with these perspectives, it has been found that social support influenced coping strategies, which in turn influenced psychological adjustment (Valentiner et al., 1994). Recently, Feeney and Collins (2015) proposed a theoretical perspective and suggested multiple pathways through which social support affects well-being. They argued that in adversities, social support makes recipients more forgiving of their failure or transgression, thus increasing self-compassion, which in turn promotes one’s long-term thriving. Based on these theoretical perspectives and empirical findings, self-compassion was hypothesized to mediate between perceived social support and life satisfaction in infertile women. Although neither the relationship between perceived social support and self-compassion nor the mediating effect of self-compassion in the relationship between perceived social support and subjective well-being has been tested in infertile individuals, findings in other populations have provided indirect evidences for them. A positive correlation between perceived social support and self-compassion was found in college students (Neely et al., 2009; Stallman et al., 2018). In addition, Toplu-Demirtaş et al. (2018) found self-compassion mediated the correlation between perceived social support from family and significant others and subjective well-being in LGB individuals, explaining the 77% of the variance in subjective well-being. Similarly, Wilson et al. (2020) found the association between perceived social support and subjective happiness, assessed by Subjective Happiness Scale (SHS; Lyubomirsky and Lepper, 1999), was partially accounted by self-compassion in college students. Hence, we hypothesized that perceived social support predicted self-compassion and self-compassion was a mediator in the relationship between perceived social support and life satisfaction in infertile women.

### The Moderating Role of Infertility Self-Efficacy

Self-efficacy refers to an individual’s beliefs about her or his capabilities to achieve goals in specific tasks (Bandura, 1977). Derived from self-efficacy, infertility self-efficacy describes an individual’s self-efficacy for dealing with infertility and its treatment (Cousineau et al., 2006). Infertility self-efficacy has been found to be related to less mental health problems including stress, depression and anxiety in infertile women in different cultures including mainland China (Cousineau et al., 2006; Galhardo et al., 2013, 2014; Arslan-Özkan et al., 2014; Fu et al., 2016; Kim et al., 2017; Khalid and Dawood, 2020). Although the relation between infertility self-efficacy and subjective well-being has not been examined in infertile individuals, it has been found that general self-efficacy was positively related to infertility specific well-being in infertile women (Batinic et al., 2017). Therefore, we speculated that infertility self-efficacy was important for infertile women’s subjective well-being.

This study hypothesized the effect of perceived social support on self-compassion in infertile women may vary depending on infertility self-efficacy. According to Bandura (1977), perceived self-efficacy determines whether coping behavior will be initiated, how much effort will be expended, and how long it will be sustained in the face of obstacles and aversive experiences. The stronger the perceived self-efficacy is, the more active the effort is. Therefore, we speculated that infertility self-efficacy affected the efforts an infertile woman spent to enhance coping behaviors. Specifically, women with higher infertility self-efficacy would make more effort to promote their coping, one manifestation of which was greater use of perceived social support to improve self-compassion. This moderating effect of infertility self-efficacy on the relationship between perceived social support and self-compassion has not been examined in the infertility population. Yet, there has been evidence of the moderating effect of self-efficacy on the relationship between social support and coping behaviors in other population groups. For example, Schwarzer and Schröder (1997) found that self-efficacy enhanced the promoting effect of social support on post-operative coping behavior “reading” in heart patients. Based on the above theoretical framework and findings, we hypothesized that infertility self-efficacy moderated the effect of perceived social support on self-compassion in infertile women.

### The Current Study

Given that the mediating mechanism for the relationship between perceived social support and life satisfaction in infertile women is still unknown, the present study aimed to test the mediating effect of self-compassion in this relationship in infertile women in mainland China. Besides, this study was the first to examine the relationship between perceived social support and life satisfaction as well as the relationship between self-compassion and life satisfaction in infertile women from mainland China. In addition, the present study attempted to provide insights into infertility self-efficacy’s moderating effect on the relationship between perceived social support and self-compassion in infertile women. This would provide more detailed information on the underlying mechanism of the relationship between perceived social support and life satisfaction. Thus, this study tested whether self-compassion mediated the relationship of perceived social support with life satisfaction and whether infertility self-efficacy moderated the relationship of perceived social support with self-compassion in infertile women undergoing treatment in mainland China.

## Materials and Methods

### Design and Procedure

An online questionnaire was used in this study to measure perceived social support, life satisfaction, self-compassion, infertility self-efficacy and some information related to infertility (age, types of infertility, and causes of infertility). The Ethics Committee of Zhengzhou University provided ethical review and approval for the present study. Infertile women undergoing treatment in a Reproductive Medicine Center of a Tertiary Hospital in Henan, China, were invited to complete the online questionnaire by a clinician.

### Participants

A total of 293 women responded to our recruitment and completed the online survey. None of the participants had a history of mental illness, substance abuse or other major health problems. Finally, data from 290 subjects were used for the main analysis. Participants ranged in age from 21 to 48 years old (*M* = 32.45, *SD* = 5.45).

### Measurements

#### Perceived Social Support

Zimet et al. (1988) developed the Multidimensional Scale of Perceived Social Support (MSPSS) to measure the perceived adequacy of social support received from family, friends, and the significant other. The MSPSS includes 12 items (e.g., “I can count on my friends when things go wrong”). Respondents report their agreement on a 7-point Likert-type scale (1 = very strongly disagree; 7 = very strongly agree). Higher total score means higher perceived social support. Jiang (1999) translated it into Chinese. Ye (2006) reported the Chinese MSPSS’s internal consistency α coefficient was 0.88 and it was negatively related to depression assessed with Center for Epidemiological Studies Depression Scale (CES-D; Radloff, 1977). Shou and Chen (2015) reported it correlated negatively to loneliness assessed with the UCLA-Loneliness Scale (UCLA-LS; Russell, 1996). The Chinese MSPSS was used in this study and showed an internal consistency reliability estimate of 0.94.

#### Life Satisfaction

The Satisfaction with Life Scale (SWLS, Diener et al., 1985) was developed to evaluate cognitive satisfaction with life. The five items (e.g., “In most ways, my life is close to my ideal”) are rated on a 7-point Likert-type scale (1 = strongly disagree; 7 = strongly agree). Higher total score indicates higher life satisfaction. The Chinese version of the SWLS was translated by Chen and Yang (2003). Yang et al. (2009) reported the scale’s internal consistency α coefficient and split-half reliability were 0.81 and 0.73, respectively, and it was positively related to self-esteem, assessed with the Rosenberg Self-esteem Scale (SES; Rosenberg, 1965), and negatively correlated with depression, assessed with the depression subscale of Symptom Check-list 90 (SCL-90; Derogatis, 1975). In this study, the Chinese SWLS was used and its internal consistency reliability estimate was 0.89.

#### Self-Compassion

The Self-Compassion Scale (SCS, Neff, 2003b) measures self-kindness (five items; e.g., “I try to be understanding and patient toward those aspects of my personality I don’t like”), self-judgment (five items; e.g., “When times are really difficult, I tend to be tough on myself”), common humanity (four items) (e.g., “When I feel inadequate in some way, I try to remind myself that feelings of inadequacy are shared by most people”), isolation (four items; e.g., “When I’m feeling down I tend to feel like most other people are probably happier than I am”), mindfulness (four items; e.g., “When something upsets me I try to keep my emotions in balance”), and over-identification (4 items; e.g., “When something upsets me I get carried away with my feelings”). All items are rated on a 5-point Likert-type scale (1 = almost never; 5 = almost always). Chen et al. (2011) reported the Chinese version of SCS’s internal consistency α coefficient and test-retest reliability were 0.84 and 0.89, respectively, and it correlated positively with self-esteem, assessed with the Rosenberg Self-esteem Scale (SES; Rosenberg, 1965), and positive affect, assessed with the Positive Affect and Negative Affect Scale (PANAS; Watson et al., 1988), and negatively with negative affect, assessed with the Positive Affect and Negative Affect Scale (PANAS; Watson et al., 1988). In this study, the Chinese SCS was used and higher total score signified higher self-compassion. Internal consistency reliability estimates for the self-kindness, self-judgment, common humanity, isolation, mindfulness, and over-identification subscales and the total scale were 0.83, 0.75, 0.72, 0.83, 0.83, 0.69, and 0.83, respectively.

#### Infertility Self-Efficacy

The Infertility Self-Efficacy Scale (ISE, Cousineau et al., 2006) was developed to assess infertile individuals’ perception of one’s capability to dealing with infertility and it’s treatment. The instrument has 16 items (e.g., “I’m confident I can keep from getting discouraged when nothing I do seems to make a difference”) which are rated on a 9-point Likert-type scale (1 = not at all confident; 9 = very confident). Higher total score means higher infertility self-efficacy. Fu et al. (2016) translated it into Chinese and reported that the scale’s Cronbach’s α and test-retest reliability were 0.94 and 0.84, respectively, and it correlated positively with positive coping style, assessed with Simplified Coping Style Questionnaire (SCSQ; Xie, 1998), and negatively with anxiety, assessed with Self-rating Anxiety Scale (SAS; Zung, 1971), and depression, assessed with Self-rating Depression Scale (SDS; Zung, 1965). Chinese version of the ISE was used to evaluate infertility self-efficacy in this study. Internal consistency reliability estimate of 0.96 was found for the scale.

### Data Analyses

As a preparatory analysis, Martensitic distances were calculated to examine multivariate outliers for all studied variables. Afterward, Harman’s one-factor test was executed to detect the common method bias of the data (Podsakoff and Organ, 1986). In the main data analysis, firstly, descriptive statistics, correlation analysis, and difference tests were performed. Secondly, PROCESS macro of Model 7 (Hayes, 2012) was executed to examine the moderated mediation model with perceived social support as the independent variable, life satisfaction as the dependent variable, self-compassion as the mediator, and infertility self-efficacy as the moderator. Additionally, we drew on the bootstrapping method (Hayes and Scharkow, 2013), which produces 95% bias-corrected confidence intervals from 5000 resamples of the data, to examine the significance of indirect effects. The effects are significant when the confidence intervals exclude zero.

## Results

### Preliminary Analyses

Martensitic distances of three participants’ data were greater than the critical value (*χ*^2^ = 16.27, *p* = 0.001, *df* = 3) (Tabachnick and Fidell, 2007) and therefore were deleted. A total of 290 participants’ data were used for the subsequent data analyses. The results of Harman’s one-factor test showed that 10 factors with eigenvalue greater than 1 were extracted by the unrotated exploratory factor analysis on all items, and the maximum factor variance interpretation rate was 30.95%. Therefore, these results suggested that there was no serious common method bias in this study (Podsakoff and Organ, 1986). These 290 participants were diagnosed with primary (*N* = 119) or secondary (*N* = 171) infertility. Their infertility was caused by female (*N* = 132), male (*N* = 28), bisexual (*N* = 69) or uncertain (*N* = 61) factors. The results of the descriptive and correlation analyses and difference tests are presented in Table 1.

**TABLE 1:** Descriptive statistics and correlations among all studied variables.

	Perceived social support	Life satisfaction	Self-compassion	Infertility self-efficacy
Life satisfaction	0.57***			
Self-compassion	0.44***	0.50***		
Infertility self-efficacy	0.45***	0.57***	0.54***	
Age	−0.02	0.02	0.11	0.10
Types of infertility (*t*)	0.85	0.50	0.61	−0.67
Causes of infertility (*F*)	0.41	1.17	0.32	1.66
*M*	68.30	24.42	88.10	112.94
*SD*	11.90	6.32	13.17	23.49

N = 290; ***p < 0.001.

### Testing for the Moderated Mediation Model

**FIGURE 1:**
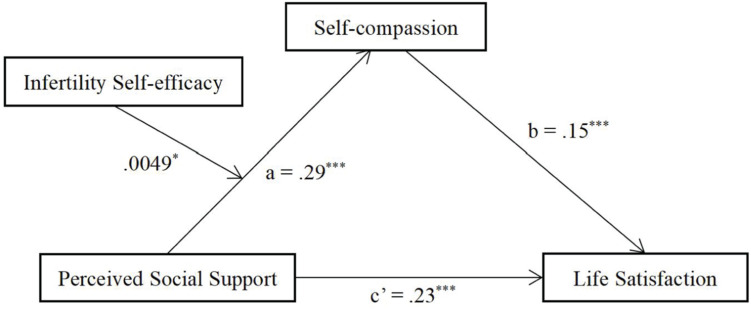
Moderated mediation model for the indirect effect of perceived social support correlated with life satisfaction via self-compassion; the relationship between perceived social support and self-compassion was moderated by infertility self-efficacy. The unstandardized coefficients are reported. *p < 0.05; ***p < 0.001.

**FIGURE 2:**
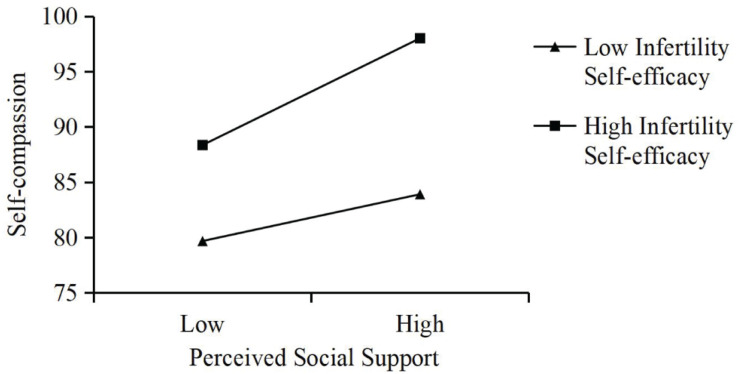
The relationship between perceived social support and self-compassion that is moderated by infertility self-efficacy.

All variables were centralized to eliminate the multicollinearity problem. Table 2 displays the results of the moderated mediation model, with perceived social support as the independent variable, life satisfaction as the dependent variable, self-compassion as the mediator, and infertility self-efficacy as the moderator. Although age was not significantly associated with life satisfaction in this study, it was positively related to infertility-related stress, which negatively affected life satisfaction (Mcquillan et al., 2007). Hence, age was treated as a covariable in the analysis to rule out its effect on the results. One standard deviation above and below the mean of infertility self-efficacy were defined as the high and low level of infertility self-efficacy. The results showed that perceived social support had an significant indirect effect on life satisfaction through self-compassion at both high (*B* = 0.06, 95% CI[0.03, 0.09]) and low (*B* = 0.03, 95% CI[0.00, 0.05]) level of infertility self-efficacy. Besides, it was found that perceived social support had an significant direct effect on life satisfaction (*B* = 0.23, *p* < 0.001). In addition, the interaction term between perceived social support and infertility self-efficacy was significantly positively related to self-compassion (*B* = 0.0049, *p* < 0.05). The moderated mediation model diagram is shown in Figure 1. The results of simple slope analysis showed that perceived social support was a stronger predictor of self-compassion when infertile women had high level of infertility self-efficacy (*B* = 0.41, *p* < 0.001) than when infertile women had low level of infertility self-efficacy (*B* = 0.18, *p* < 0.05) (Figure 2).

**TABLE 2:** Testing the moderated mediation model.

Predictors	Model 1 (Self-compassion)			Model 2 (Life satisfaction)		
	*B*	*SE*	*t*	*B*	*SE*	*t*
Perceived social support	0.29	0.06	4.93***	0.23	0.03	8.45***
Infertility self-efficacy	0.24	0.03	8.06***			
Social support × Infertility self-efficacy	0.0049	0.00	2.21*			
Self-compassion				0.15	0.02	6.03***
Age	0.21	0.12	1.78	−0.00	0.05	−0.09
*R*^2^	0.36			0.40		
*F*	39.87***			63.67***		

N = 290; Each column is a regression model that predicts the criterion at the top of the column. *p < 0.05; ***p < 0.001.

## Discussion

The present study was the first to test the mediating effect of self-compassion in the relationship between perceived social support and life satisfaction in infertile women, and the results supported the mediating effect of self-compassion. The finding was consistent with previous findings. For example, self-compassion mediated the relationship between perceived social support and subjective well-being in LGB individuals and the relationship between perceived social support and subjective happiness in college students (Toplu-Demirtaş et al., 2018; Wilson et al., 2020). This finding implied that as an emotion-approached coping strategy, infertile women’s self-compassion may be enhanced by perceived social support, which was thought to be a source of coping assistance (Thoits, 1986). There may be different ways in which infertile women’s self-compassion is enhanced by perceived social support. For example, infertile women’s perceived social support may cause them to forgive their own shortcomings, leading to increased self-compassion (Feeney and Collins, 2015). In addition, according to Ullman and Filipas (2001), perceived social support from close others includes care and empathy. Therefore, infertile women may learn care and empathy toward themselves from social interaction with close others, leading to higher level of self-compassion (Maheux and Price, 2016). Because of improved self-compassion, infertile women may have a more positive attitude toward a life of infertility. For instance, they may think about the benefits of having a child-free life, such as more free time, saving more money and enjoying leisure-time interests or hobbies (Tiu et al., 2018). This positive attitude toward infertile life may result in a high life satisfaction.

Besides, this study examined whether infertility self-efficacy moderated the relationship between perceived social support and self-compassion. As expected, infertility self-efficacy displayed a moderating effect on the relationship between perceived social support and self-compassion. In particular, perceived social support had a larger positive predictive effect on self-compassion when infertile women had higher infertility self-efficacy. The finding was in line with previous findings. For instance, perceived social support had a larger positive predictive effect on post-operative coping behavior “reading” when heart patients had higher self-efficacy Schwarzer and Schröder (1997). This finding suggested that higher infertility self-efficacy enhances the effect of perceived social support on self-compassion. This supported our hypothesis that infertile women with higher infertility self-efficacy make more effort to facilitate their coping with infertility, as manifested by greater use of perceived social support to promote self-compassion in the present study.

These findings have implications for the practice of psychological counseling aimed at improving infertile women’s life satisfaction, especially those who are undergoing treatment. Firstly, these findings emphasized the importance of social support in maintaining life satisfaction in infertile women. Given that infertile women often do not receive adequate support from conventional sources including intimate others, medical professionals, and online sources (Cwiek et al., 2009; High and Steuber, 2014), perceived support through counseling seems to be necessary to ensure infertile women’s life satisfaction. Therefore, infertile women should be advised and encouraged to receive psychological counseling. Secondly, our findings demonstrated that perceived social support was associated with life satisfaction through self-compassion in infertile women. Hence, providing support to promote self-compassion and thus increase life satisfaction is a good counseling strategy for infertile women clients. Finally, self-compassion training has been shown to improve psychological well-being (i.e., autonomy, environmental mastery, personal growth, positive communication with others, purpose in life, and self-acceptance) (Afshani et al., 2019), therefore, counselors can try to improve infertile women clients’ life satisfaction by teaching them to practice self-compassion.

### Limitations

Firstly, the study did not collect more demographic information to rule out their influence on the results. Future research should give adequate attention to this issue. Secondly, the participants in this study were a single sample from a reproductive medicine center in a Chinese hospital. Future research should examine whether these findings can be verified in other diverse samples. Thirdly, this study was a cross-sectional study, which prevented us from drawing causal conclusions. Perceived social support, self-compassion, and life satisfaction could be related in bidirectional ways. Future research should use longitudinal or experimental studies to verify our findings and make causal conclusions. Fourthly, the data collected in this study included the names of the participants and could be seen by their doctors. As a result, it was possible that to meet the expectations of doctors and manage their own image, the participants made answers inconsistent with their true feelings in their self-reports, which led to social desirability bias. In future studies, more objective and accurate measurements could be used to verify our findings. Finally, this study did not directly investigate how the infertility and the related stress could be alleviated by the protective factors such as social support and self-compassion, as social support rather than infertility-related stress was the independent variable in this study.

## Data Availability Statement

The raw data supporting the conclusions of this article will be made available by the authors, without undue reservation.

## Ethics Statement

The studies involving human participants were reviewed and approved by the Ethics Committee of Zhengzhou University. Written informed consent for participation was not required for this study in accordance with the National Legislation and the Institutional Requirements.

## Author Contributions

WG completed the research design. WG, YG, and RZ completed the manuscript writing. XC collected the data. All authors contributed to the article and approved the submitted version.

## Conflict of Interest

The authors declare that the research was conducted in the absence of any commercial or financial relationships that could be construed as a potential conflict of interest.
